# *Porphyromonas gingivalis* exacerbates experimental autoimmune encephalomyelitis by driving Th1 differentiation via ZAP70/NF-κB signaling

**DOI:** 10.3389/fimmu.2025.1549102

**Published:** 2025-03-18

**Authors:** Dong Dai, Guoqin Cao, Shengyuan Huang, Min Xu, Jilei Wang, Xue Han, Qiuying Ma, Jiang Lin

**Affiliations:** ^1^ Department of Stomatology, Beijing Tongren Hospital, Capital Medical University, Beijing, China; ^2^ Department of Neurology, Beijing Tongren Hospital, Capital Medical University, Beijing, China

**Keywords:** Porphyromonas gingivalis, periodontitis, experimental autoimmune encephalomyelitis, Th1 cell, NF-κB signaling pathway, multiple sclerosis

## Abstract

**Background:**

Multiple sclerosis (MS) is characterized by chronic inflammation and demyelination within the central nervous system (CNS), primarily driven by the abnormal activation of the peripheral immune system, notably Th1 cells. As the principal pathogen in periodontitis, *Porphyromonas gingivalis* (*P. gingivalis*) is linked to an increased risk of multiple sclerosis progression; however, its role in central nervous system inflammation remains unclear. In this study, we aimed to determine whether *P. gingivalis* promotes peripheral Th1 cell differentiation via the ZAP70/NF-κB signaling pathway, thereby exacerbating experimental autoimmune encephalomyelitis(EAE), a model of multiple sclerosis.

**Methods:**

C57BL/6J mice were randomly divided into healthy control, periodontitis, EAE, and periodontitis with EAE group. Neurological function was assessed using Weaver’s score. Histopathology (H&E, LFB staining) and Evans blue dye leakage evaluated inflammation, demyelination, and blood-brain barrier(BBB)permeability. Th1 and Th17 cells were quantified by flow cytometry, while immunofluorescence staining was performed to analyze Claudin-5, IFN-γ ^+^CD4^+^ T -positive cell and IL-17^+^CD4^+^-positive cell expression. Western blotting measured NF-κB and related protein expression. Reference-based mRNA sequencing analysis and Kyoto encyclopedia of genes and genomes (KEGG) pathway enrichment was conducted to identify differential gene expression and pathway enrichment.

**Results:**

In mice with experimental autoimmune encephalomyelitis, *P. gingivalis* infection significantly elevated Th1 cell proportions in the peripheral blood, increased interferon-gamma expression, and exacerbated central nervous system inflammation and demyelination by enhancing blood–brain barrier permeability. The infection also activated the ZAP70/NF-κB pathway, essential for peripheral Th1 differentiation, as evidenced by p65 nuclear translocation and significant upregulation of Th1-related genes, including those of the transcription factor *Tbx21* and interleukin-12 receptors. *In vitro*, *P. gingivalis* lipopolysaccharide (LPS) stimulated Th1 differentiation via ZAP70/NF-κB, which was effectively blocked by pathway inhibitors, reducing Th1 cells and pro-inflammatory factors.

**Discussion:**

Our findings elucidate, for the first time, how *P. gingivalis* infection promotes central nervous system inflammation by driving Th1 cell differentiation via peripheral ZAP70/NF-κB pathway activation. This highlights *P. gingivalis* as a local periodontitis pathogen and significant contributor to neuroinflammation, providing new insights into the pathogenesis of multiple sclerosis and identifying promising targets for immunomodulatory therapeutic strategies.

## 1 Introduction

Multiple sclerosis (MS) is a chronic autoimmune disease of the central nervous system (CNS) marked by inflammation and demyelination (1, 2). It is the leading non-traumatic cause of disability in young adults (3). The primary pathogenesis of MS is predominantly characterized by the activation of peripheral CD4^+^ T helper cells (2), particularly of the Th1 and Th17 subsets (4–6), which migrate to the CNS and initiate local immune responses. Th1 cells specifically target myelin antigens within the CNS, directly damage myelin, and increase the permeability of the blood–brain barrier (BBB) through interferon-gamma (IFN-γ) secretion (7). Subsequently, more immune cells are recruited to the CNS, resulting in elevated inflammation and ultimately leading to demyelination and neuronal damage. Experimental autoimmune encephalomyelitis (EAE) is a CD4+ T cell-mediated neuroinflammatory disease induced by myelin antigens, characterized by CNS mononuclear infiltration and demyelination, resembling multiple sclerosis (MS) both pathologically and immunologically. With clinical manifestations such as limb paralysis and ataxia, EAE is widely recognized as the gold-standard animal model for MS (8).

Periodontitis, a chronic inflammatory disease caused by periodontal pathogens, leads to the destruction of periodontal supporting tissues and is one of the most prevalent oral diseases (9). *Porphyromonas gingivalis*, the primary pathogen of periodontitis, is highly abundant in patients with MS, and research indicates that periodontitis increases MS susceptibility (10). Furthermore, oral infection of *P. gingivalis* exacerbates the clinical symptoms of EAE (11), possibly due to the inflammation-related immune responses common between MS and periodontitis.

*P. gingivalis* and its virulence factors can enter the systemic circulation, increasing the proportion of CD4^+^ T cells in the peripheral immune system, particularly pro-inflammatory Th1 cells (12). Th1 differentiation is regulated by several signaling pathways, including JAK-STAT, MAPK, and NF-κB (13). *P. gingivalis* lipopolysaccharides (LPS) have been demonstrated to activate the NF-κB pathway in human gingival epithelial and dental pulp stem cells (14). NF-κB promotes Th1 differentiation by regulating the expressions of transcription factor T-bet and IL-12 receptor (15). However, the specific upstream regulatory molecules of NF-κB in Th1 differentiation remain to be fully elucidated. Given that the NF-κB signaling pathway plays a pivotal role in regulating Th1 differentiation and inflammatory responses, it has become a classical immune checkpoint in immunotherapy research. Therefore, we hypothesized that periodontitis and MS may interact via the NF-κB-Th1 pathway. However, the precise pathological interactions and their molecular mechanisms remain unclear.

In this study, we aimed to investigate whether *P. gingivalis* mediates Th1 cell differentiation to exacerbate EAE pathology and to explore the underlying mechanism. The findings provide novel insights into the relationship between periodontitis and MS, and reveal a theoretical basis for developing new therapeutic strategies for MS.

## 2 Materials and methods

### 2.1 Animal

Thirty-two C57 BL/6J mice (6–8 weeks old, female) were purchased from Beijing Vital River Laboratory Animal Technology Co. Ltd to be used in the study. All mice were raised at the Animal Experimental Center of Capital Medical University under pathogen-free conditions and acclimatized for at least 2 weeks prior to immunization. All experimental procedures were carried out in accordance with the guidelines of the Institutional Animal Care and Use Committee. The protocol was authorized by the Animal Experimental Ethics Committee of Beijing MDKN. The mice were randomly divided into four groups (n = 8): healthy control (CON group), periodontitis (P group), EAE (EAE group), and periodontitis with EAE (EAE+P group).

### 2.2 Bacterial culture

*P. gingivalis* (strain W83, BNCC) was cultured on enriched sterile blood agar consisting of 3.7% brain heart infusion (BHI) agar, 2% agarose, 0.05% cysteine, 0.0005% hemin, 0.0001% menadione, and 5%–7% defibrinated sheep blood, under anaerobic conditions generated by an AnaeroPack. After 4–5 days of incubation, a single colony was transferred to BHI broth containing 3.7% BHI, 0.0005% hemin, 0.0001% menadione, and 0.05% cysteine, and cultured for an additional 48 h.

### 2.3 Establishment of an EAE model

To induce EAE, a 10 mg/mL solution of myelin oligodendrocyte glycoprotein (MOG)35-55 peptide (QYAOBIO) was emulsified with complete Freund’s adjuvant (16) (CFA, F5881,Sigma-Aldrich Corp.) at a concentration of 5 mg/mL in a 1:1 volume ratio. As described previously (6), 100 µL of the emulsion was injected subcutaneously at four sites on the lower back of the mice, with 25 µL administered per site in a single immunization. Immediately after immunization (Day 0) and within 48 h, each mouse received an intraperitoneal injection of 200 µL of pertussis toxin (PTX, GC17532, GlpBio) at a concentration of 1 µg/mL. Mice were randomly assigned to the experimental group using a random number table. Starting on the day of immunization, the mice were scored daily for neurological function using a 15-point scale known as the Weaver’s score (17). This score differentiates between individual limb impairments rather than grouping the fore and hind limbs together, allowing for a better characterization of disease progression. The total score ranges from 0 to 15 and is the sum of the disease status scores for the tail (0–2) and all four limbs (0–3 for each limb).

For the tail assessment:

0 indicates no symptoms.

1 represents reduced tail tone or partial paralysis of the tail.

2 indicates full paralysis of the tail.

For limb function:

0 is given for no symptoms.

1 is assigned when the mouse exhibits an unstable gait.

2 denotes mild limb paralysis, where dragging is observed during walking.

3 is for severe limb paralysis, where limbs are outwardly flipped during walking.

Thus, based on this scoring system, a fully tetraplegic mouse would receive a score of 14 and a deceased mouse would receive a score of 15.

### 2.4 Establishment of a periodontitis model

To establish periodontitis in mice, *P. gingivalis*-soaked 6-0 silks were placed around the maxillary second molars on day 7 before EAE immunization, followed by daily oral inoculation of 100 µL *P. gingivalis* suspension (1 × 10^9^ CFU/mL) for 24 consecutive days. Successful establishment of the periodontitis model was confirmed by consistent clinical signs of inflammation and validated through histological evidence of inflammatory cell infiltration and alveolar bone resorption.

### 2.5 Histopathology

After anesthesia, the mice were transcardially perfused with 4% paraformaldehyde. The spinal cords were collected, dehydrated in a graded series of ethanol (70%, 80%, 95%, and 100%), cleared in xylene, and embedded in paraffin. Next, 3 μm sections of these tissues were prepared continuously for hematoxylin and eosin (H&E) and Luxol fast blue (LFB) stainings to determine the severity of neuroinflammation and demyelination, respectively. The severity of inflammation was scored as follows (18):0, no inflammation, 1, cellular infiltrate only in the perivascular areas and meninges, 2, mild cellular infiltrate in parenchyma(1-20/section);3,moderate cellular infiltrate in parenchyma (21-100/section);4, severe cellular infiltrate in parenchyma(>100/section).

The level of demyelination was evaluated by calculating the ratio of the residual myelin area within the injury center of the spinal cord. Following LFB staining, the residual myelin area was measured using the ImageJ image analysis software(National institutes of Health, Bethesda, MD, USA). The myelin retention rate, expressed as the percentage of the residual myelin area relative to the total area of the injury center, provided a quantitative assessment of the severity of demyelination.

### 2.6 Immunofluorescent staining

Paraffin sections were deparaffinized and rehydrated using a graded series of xylene and alcohol. For antigen retrieval, the sections were boiled in Tris-EDTA solution (pH = 9.0) for 15 min. The primary antibodies used were: rabbit anti-mouse Claudin-5 (GB111621,Servicebio,1:200), rat anti-mouse CD4 (GB13064, Servicebio,1:100), rabbit anti-mouse IFN-γ (GB11109,Servicebio,1:200), and rat anti-mouse IL-17 (GB13073, Servicebio,1:100). For visualization, the secondary antibodies Alexa Fluor 488 goat anti-rat IgG (GB25301, Servicebio,1:500) and Alexa Fluor 647 goat anti-rabbit IgG (GB25303, Servicebio,1:500) were used to detect the rat- and rabbit-derived primary antibodies, respectively. DAPI was used as a nuclear counterstain following secondary antibody incubation. Fluorescent images were captured using a Leica TCS SP5 confocal system with a 63× objective lens, optimizing laser wavelengths for each fluorophore to ensure clear signal detection.

### 2.7 Evans blue dye leakage assay

A 2% Evans blue (EB) solution (2 g of dye dissolved in 100 mL of double-distilled water) was prepared. After anesthetizing the mice, 4 mL/kg of the dye solution was injected through the tail vein and allowed to remain for 0.5–1 h. Next, cardiac perfusion was performed as follows: 0.9% NaCl solution (containing heparin) was slowly injected into the apex of the heart, and the right atrium was clipped until the effluent was clarified. The brain tissue was removed, weighed, and ground thoroughly using a homogenizer following the addition of 1 mL PBS. The supernatant was collected by centrifugation at 1000 × g for 15 min, treated with an equal amount of trichloroacetic acid, and refrigerated overnight. Finally, the absorbance was measured at 620 nm using a spectrophotometer and the dye concentration was calculated from the standard curve(Thermo Scientific NanoDrop).

### 2.8 Flow cytometry

Flow cytometry was used to detect the proportion of Th1 (CD4^+^IFN-γ^+^) and Th17 (CD4^+^IL-17A^+^) cells in the peripheral blood and splenic lymphocytes of mice. The blood and lymphocytes were first incubated with a Cell Activation Cocktail (with Brefeldin A; 423304,BioLegend,1:500) for 6 h at 37°C. Following this, the cells were collected by washing with PBS and stained for viability using the Zombie NIR™ Fixable Viability Kit (423105,BioLegend, item no.) for 15 min, protected from light. Subsequently, the cells were washed again with PBS and resuspended in the cell staining buffer. To ensure accurate detection, the cells were stained with Brilliant Violet 421™ Anti-Mouse CD4 Antibody (100443,BioLegend, item no.), Alexa Fluor^®^ 488 Anti-Mouse IFN-γ Antibody (505815,BioLegend, item no.), and PE Anti-Mouse IL-17A Antibody (506903,BioLegend. Item No.) for 20 min, protected from light. The stained cells were washed, fixed with PBS, and permeabilized to ensure the detection of Th1 and Th17 cells. Data acquisition was performed using a Cytek NL-CLC3000 flow cytometer (Cytek, USA) and a minimum of 10,000 CD4^+^ cells were collected using SpectroFlo^®^ CLC software. For analysis, FSC-A vs FSC-H was used to exclude adherent cells, FSC-A vs SSC-A was used to exclude particles, and Zombie NIR negative gates were set to label live cells. CD4^+^ Th cells were circled for selection before analyzing IFN-γ^+^ Th1 and IL-17A^+^ Th17 cells.

### 2.9 Reference-based mRNA sequencing analysis

T cells isolated from the splenic lymphocytes of mice in the EAE, and EAE+P groups (n = 4) were collected at 17 dpi. These samples were immediately frozen in liquid nitrogen at −80°C. RNA was extracted using the TRIzol method (Invitrogen, USA), treated with DNase I (Takara, Japan), and evaluated for degradation and concentration using agarose gel electrophoresis, an Agilent 2100 Bioanalyzer, and a NanoDrop spectrophotometer. For library preparation, 1.5 μg of RNA per sample was processed using the NEBNext^®^ Ultra™ RNA Library Prep Kit (NEB, USA), including mRNA purification, fragmentation, and synthesis of first- and second-strand cDNA. Adaptors were ligated and cDNA fragments of 200–250 bp were selected using the AMPure XP system (Beckman Coulter, USA) before amplification and sequencing on the Illumina Novaseq 6000 platform. Data analyses included quality control to remove low-quality reads, adapter sequences, and poly-N sequences. Clean reads were aligned to the reference genome using STAR and SNPs were identified using Picard and GATK. Gene expression levels were quantified using HTSeq, and differential expression analysis was performed using DESeq or DEGseq, depending on the availability of replicates. Gene Ontology (GO) enrichment analysis was conducted using the GOseq R package, whereas Kyoto encyclopedia of genes and genomes (KEGG) pathway enrichment was performed using KOBAS. Finally, the protein-protein interactions of the differentially expressed genes were predicted using STRING and visualized in Cytoscape.

### 2.10 Cell culture

To investigate the effect of *P. gingivalis* LPS on the promotion of peripheral Th1 cell differentiation, splenic lymphocytes were divided into four experimental groups: (1) control (Con), untreated splenic lymphocytes; (2) LPS, splenic lymphocytes stimulated with *P. gingivalis* LPS (SMB00610,Sigma–Aldrich, catalog no. 100 ng/mL) for 6 h; (3) NF-κB inhibitor + LPS: splenic lymphocytes pretreated with NF-κB inhibitor PDTC (A462174,Ambeed, catalog no.25 µM) for 30 min, followed by LPS stimulation (100 ng/mL) for 6 h; (4) ZAP70 inhibitor + LPS: splenic lymphocytes pretreated with ZAP70 inhibitor ZAP-180013 (A1359461,Ambeed, catalog no. 10 μM) for 30 min, followed by LPS stimulation (100 ng/mL) for 6 h. Following stimulation, CD4^+^ T cells were isolated from the treated splenic lymphocytes using a CD4^+^ T Cell Isolation Kit (130-104-454,Miltenyi Biotec, catalog no.) via negative selection.

### 2.11 Elisa

The IFN-γ concentration in splenic lymphocyte supernatants was measured using a mouse IFN-γ ELISA kit (JM-02465M1, Lot No. 202403,Jingmei, catalog no.). Briefly, 10 µL of sample, 40 µL of diluent, and 100 µL of HRP-conjugated antibody were added to anti-IFN-γ-coated wells and incubated at 37°C for 60 min. After washing, 50 µL each of tetramethylbenzidine substrates A and B were added, followed by incubation in the dark at 37°C for 15 min. The reaction was stopped using 50 µL of stop solution, and OD was measured at 450 nm using a Thermo microplate reader (Model: MK3).

### 2.12 qRT-PCR

Spleen lymphocytes were isolated from mice and T cells were extracted for qRT-PCR analysis of *Tnf,Il12b, Tbx21,Ifng, Rela* and *Nfkbia* mRNA expression. Total RNA was extracted using the RNAiso Plus reagent (9109,Takara Bio, Japan, catalog no.), and its concentration and purity were assessed using a spectrophotometer. cDNA was synthesized using the RT Easy™ II Kit (RT-01022/01023,FOREGENE, China, catalog no.). qRT-PCR was performed on the SLAN-96S PCR system (Shanghai Hongshi, China) in a 10 μL reaction volume containing SYBR qPCR SuperMix Plus (M00041,Sichuan Lanyun, catalog no.) and the respective primers. The protocol consisted of an initial denaturation at 95°C for 2 min, followed by 40 cycles, and a final melt curve analysis. The primers (synthesized by Chengdu Qingke Biotechnology Co.) used were as follows: Gene expression levels were calculated using the 2^-ΔΔCt method, with Actb as the internal control. The primers sequences were shown on **Table 1**.

**Table 1:** Sequences for RT-PCR.

gene	sequence (5' - 3')	
*Rela*	F	ACCTATGAGACCTTCAAGAGT
	R	GGTAGGCACAGCAATACG
*Tnf*	F	ACTGAACTTCGGGGTGATCG
	R	TGGTGGTTTGTGAGTGTGAGG
*Il12b*	F	CGCCACACAAATGGATGCAA
	R	TGTGTCCTGAGGTAGCCGTA
*Ifng*	F	GAGGTCAACAACCCACAGGT
	R	GGGACAATCTCTTCCCCACC
*Tbx21*	F	ATTGGTTGGAGAGGAAGCGG
	R	GCACCAGGTTCGTGACTGTA
*Nfkbia*	F	CCTGACCTGGTTTCGCTCTT
	R	AGGGGGAGTAGCCTTGGTAG
*Actb*	F	CTGTGCTATGTTGCTCTA
	R	GTTGCCAATAGTGATGAC

### 2.13 Western blot

A total of 1 × 10^8^ T cells isolated from splenic lymphocytes were lysed using an ultrasonic disintegrator (JY92-IIN; Ningbo Xinzhi Biotechnology Co., Ltd., China) at 30–35 kHz and 50 μA for 2 min with constant stirring. Total protein concentration was determined using the Bradford method. Proteins were precipitated with acetone, solubilized, boiled for 5 min, and stored at −70°C. For nuclear protein extraction, cells were washed with PBS, resuspended in 100 μL RIPA lysis buffer with 5 μL PMSF (1:100), scraped, and kept on ice. Protein quantification was performed using the BCA protein assay kit (Solarbio, China). Samples, including nuclear extracts and total proteins, were resolved on a 10% polyacrylamide gel using SDS-PAGE and transferred to a PVDF membrane (ISEQ00010,Merck Millipore) using a Trans-Blot chamber (Bio-Rad, USA). The membrane was blocked with 5% BSA in TBST for 1.5 h at 37°C, then incubated for 2 h at 37°C with primary antibodies diluted in TBST with 5% BSA, including: NF-κB p65 (Rabbit anti-NF-κB p65, 32536,Abcam; 1:1000), phospho-NF-κB p65 (Ser276/Ser536,76302, Abcam; 1:1000), phospho-IkBα (Ser176/180; AP0707,ABclonal; 1:1000), IkBα (Rabbit anti-IkBα, 4814S,Cell Signaling Technology; 1:1000), and housekeeping proteins β-actin (total protein control, Rabbit anti-β-actin, 700068-8F10,Zheng Neng; 1:5000) and Histone H3 (nuclear control, Mouse anti-H3, 250182, Zheng Neng; 1:1000). After washing, the membrane was incubated with a biotinylated goat anti-rabbit secondary antibody [Biotin-SP AffiniPure Goat Anti-Rabbit IgG (H + L), Jackson ImmunoResearch, USA; 1:200,000] for 1 h at 37°C, followed by 0.1 µg/mL peroxidase-conjugated streptavidin (Jackson ImmunoResearch, USA). Detection was performed using ECL chemiluminescence reagents (Amersham/GE Healthcare), and images were captured with a Vilber Lourmat TFX-35 WL transilluminator (France). Protein band intensities were quantified using ImageJ software (NIH, USA), and normalized against housekeeping proteins (β-actin for total protein and Histone H3 for nuclear proteins) to control for variations in protein loading.

### 2.14 Statistical analysis

Statistical analyses were performed using GraphPad Prism 9.0 and presented as mean ± SD. One-way ANOVA with Bonferroni’s *post-hoc* test was used to analyze the data; p-values <0.05 were considered statistically significant.

## 3 Results

### 3.1 Periodontitis significantly exacerbated the pathogenesis of EAE

#### 3.1.1 Periodontitis significantly exacerbated the clinical symptoms of EAE

**Figure 1:**
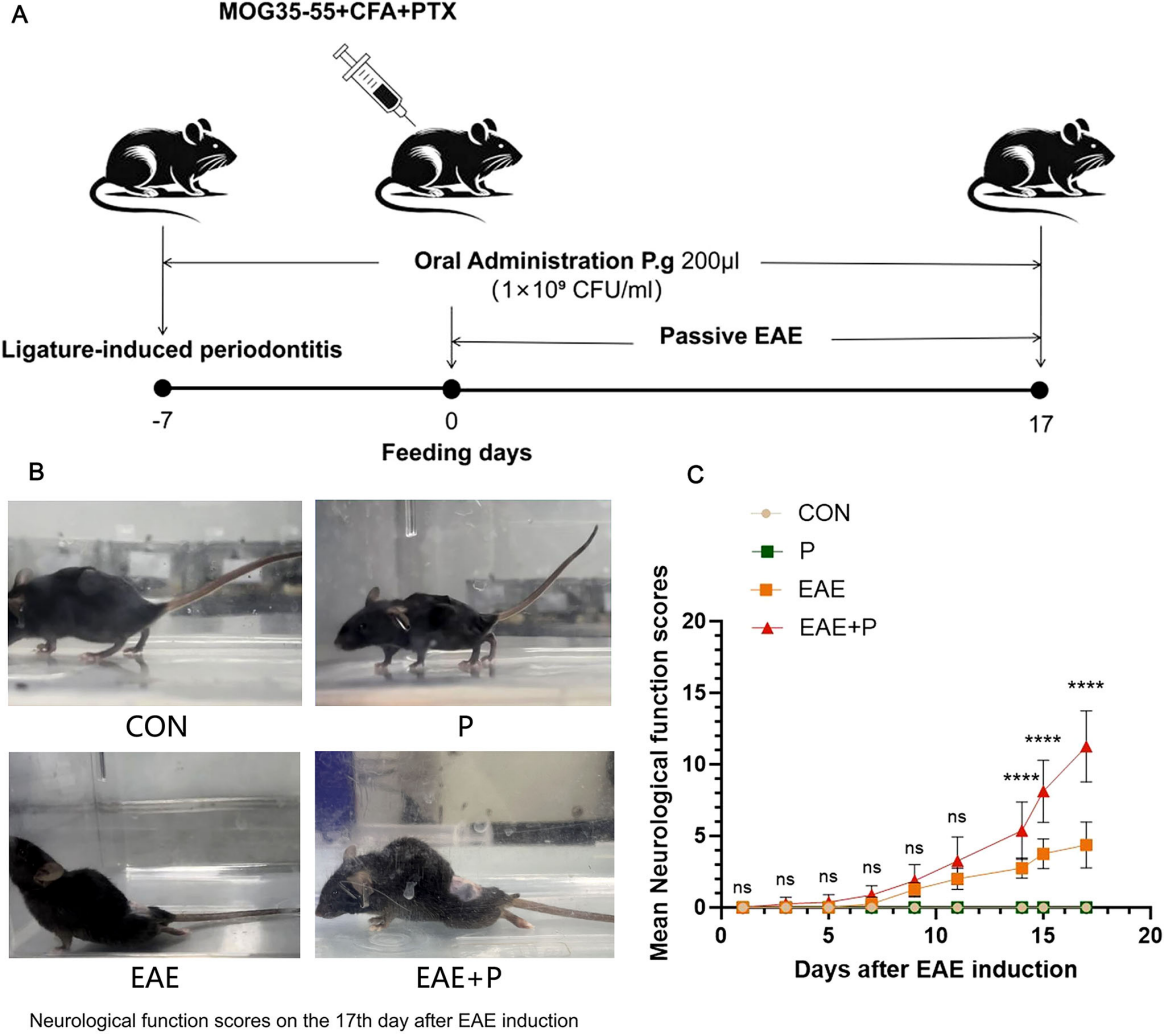
Periodontitis significantly exacerbated the clinical symptoms of the experimental autoimmune encephalomyelitis(EAE). **(A)** Experimental design: To establish periodontitis in mice, *P. gingivalis*-soaked 6-0 silks were placed around the maxillary second molars 7 days before induction, followed by oral administration of *P. gingivalis* for 24 days. Subsequently, the mouse were immunized with MOG_35-55_ peptide, CFA, and PTX on day 0, and observed until 17 dpi. **(B)** Representative images of neurological function from each group on 17 dpi. **(C)** Mice from each group were monitored every day for clinical signs of the disease from 0 to 17 dpi. Data are expressed as mean ± SD from each group (n = 8); ****p < 0.0001.

The periodontitis mice model was successfully established by *P. gingivalis* infection and ligation which was confirmed by the observation of inflammatory cell infiltration and alveolar bone resorption via H&E staining. (**Supplementary Figure S1**). Neurological function scores (13) were used to evaluate the effect of periodontitis on clinical symptoms in EAE mice, with daily monitoring of clinical scores from day 0 to 17 following EAE induction to assess the impact on disease pathogenesis(**Figures 1A, B**). After immunization, the mice in the EAE group showed neurological signs at 7 dpi, and the score peaked at 17 dpi. In terms of disease onset, no significant difference was observed between the EAE+P group and the EAE group. Neurological function scores significantly increased in the EAE+P group compared to those in the EAE group after day 14 (p < 0.0001) (**Figure 1C**).

#### 3.1.2 Periodontitis significantly exacerbated the pathological changes in EAE

**Figure 2:**
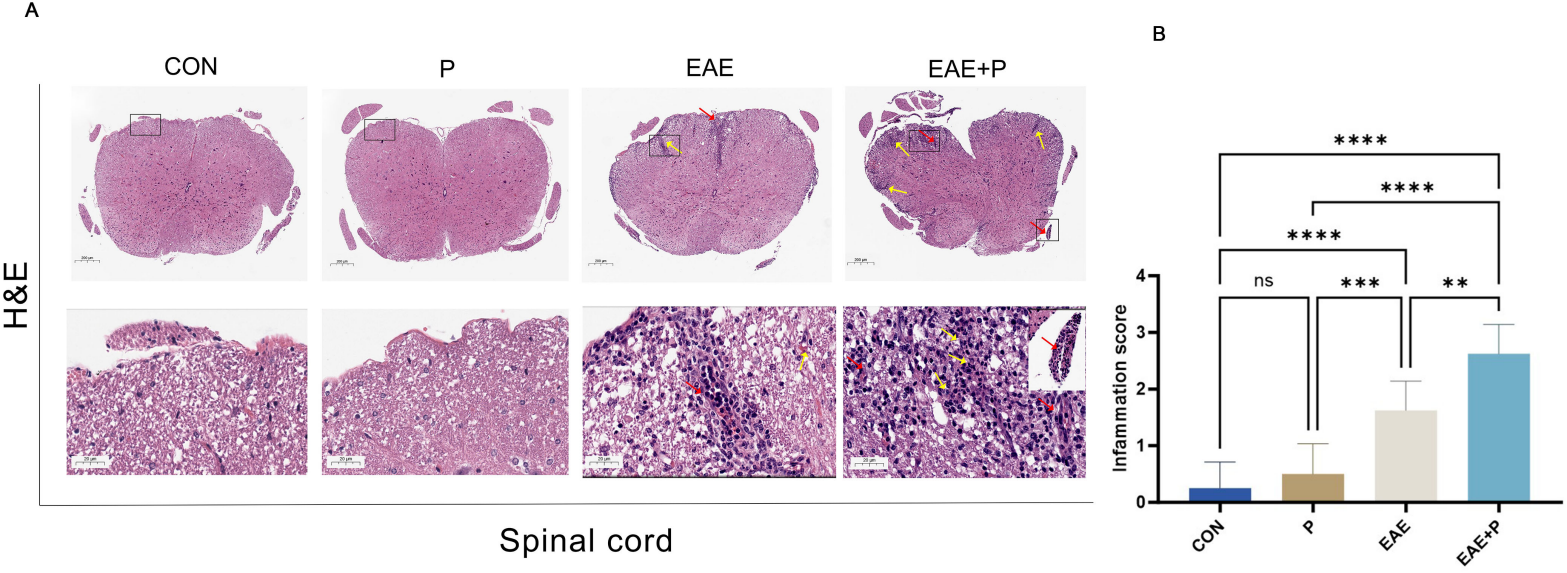
Periodontitis significantly exacerbated pathological changes in EAE. Inflammatory symptoms in the spinal cord: **(A)** Representative H&E staining images of the spinal cord from each group (n = 5). Black boxed areas are magnified in the following row, with yellow arrows indicating inflammatory cell infiltration and red arrows indicating perivascular cuffing. **(B)** Statistical analysis of inflammation scores for each group. Data are expressed as mean ± SD; **p < 0.01, ***p < 0.001, and ****p < 0.0001,NS p ≥ 0.05.

The most prominent pathological features of MS and EAE were demyelination and inflammatory cell infiltration in the CNS. H&E and LFB staining were performed to assess the effects of periodontitis on immune cell infiltration and myelin degradation in the spinal cord. H&E staining revealed a large number of mononuclear cells in the spinal cord of the EAE model group, especially around microvessels, forming “sleeve-like” structures. Compared with the EAE group, mononuclear cells were notably increased in the EAE+P group (**Figure 2A**), and the inflammation scores were significantly higher (p < 0.01) (**Figure 2B**).

**Figure 3:**
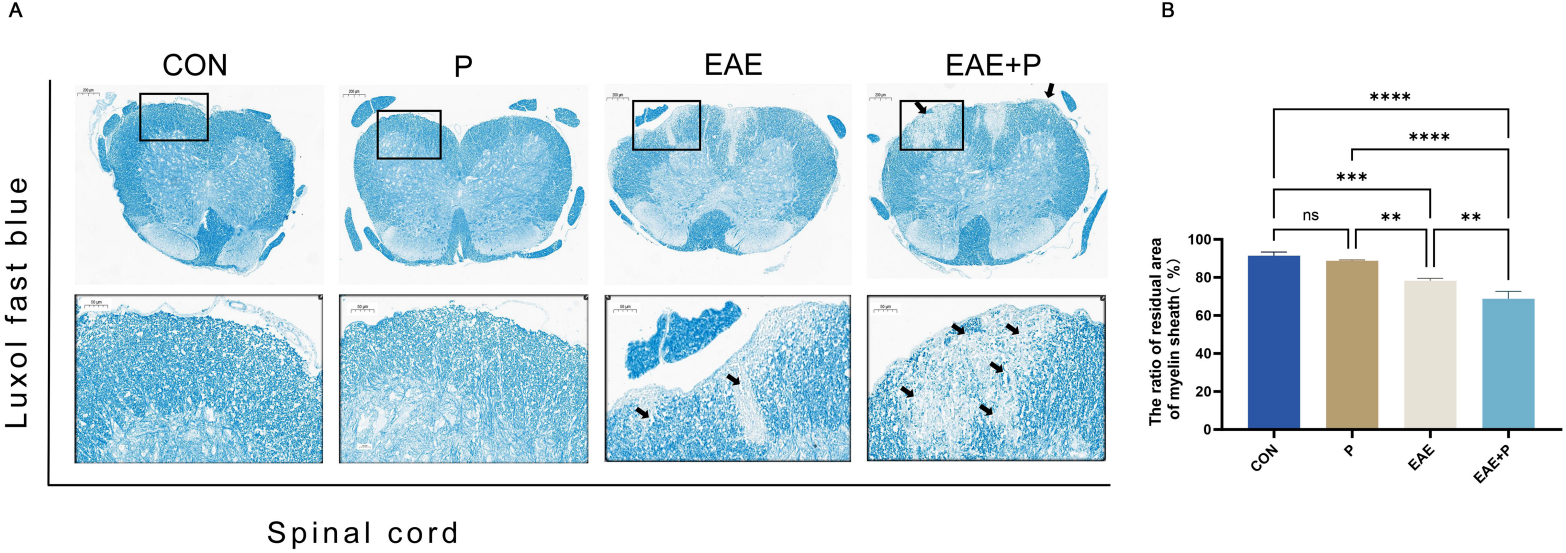
Demyelination symptoms in the spinal cord: **(A)** Representative LFB staining images of the spinal cord from each group (n = 5). Black boxed areas are magnified in the following row, with black arrows indicating the areas of myelin loss. **(B)** Statistical analysis of the ratio of the residual area of myelin sheath of the spinal cord for each group. Data are expressed as mean ± SD. **p < 0.01, ***p < 0.001, and ****p < 0.0001, NS p ≥ 0.05.

Furthermore, the orderly and complete structure of the spinal cord was evenly colored in normal mice after LFB staining, whereas an obvious diffuse demyelination area accompanied by vacuole-like changes appeared in the spinal cord of EAE mice. The demyelinated structures in the spinal cord were not observed in the P group(**Figure 3A**). The residual area of the myelin sheath of the spinal cord was lower in the EAE+P group than in the EAE group (p < 0.05) (**Figure 3B**). These results indicate that periodontitis significantly exacerbated the demyelination in EAE mice.

### 3.2 Periodontitis significantly increased the proportion of Th1 cells in the peripheral blood and splenic lymphocytes of EAE mice

#### 3.2.1 Periodontitis significantly increased the proportion of Th1 cells in the peripheral blood of EAE mice

**Figure 4:**
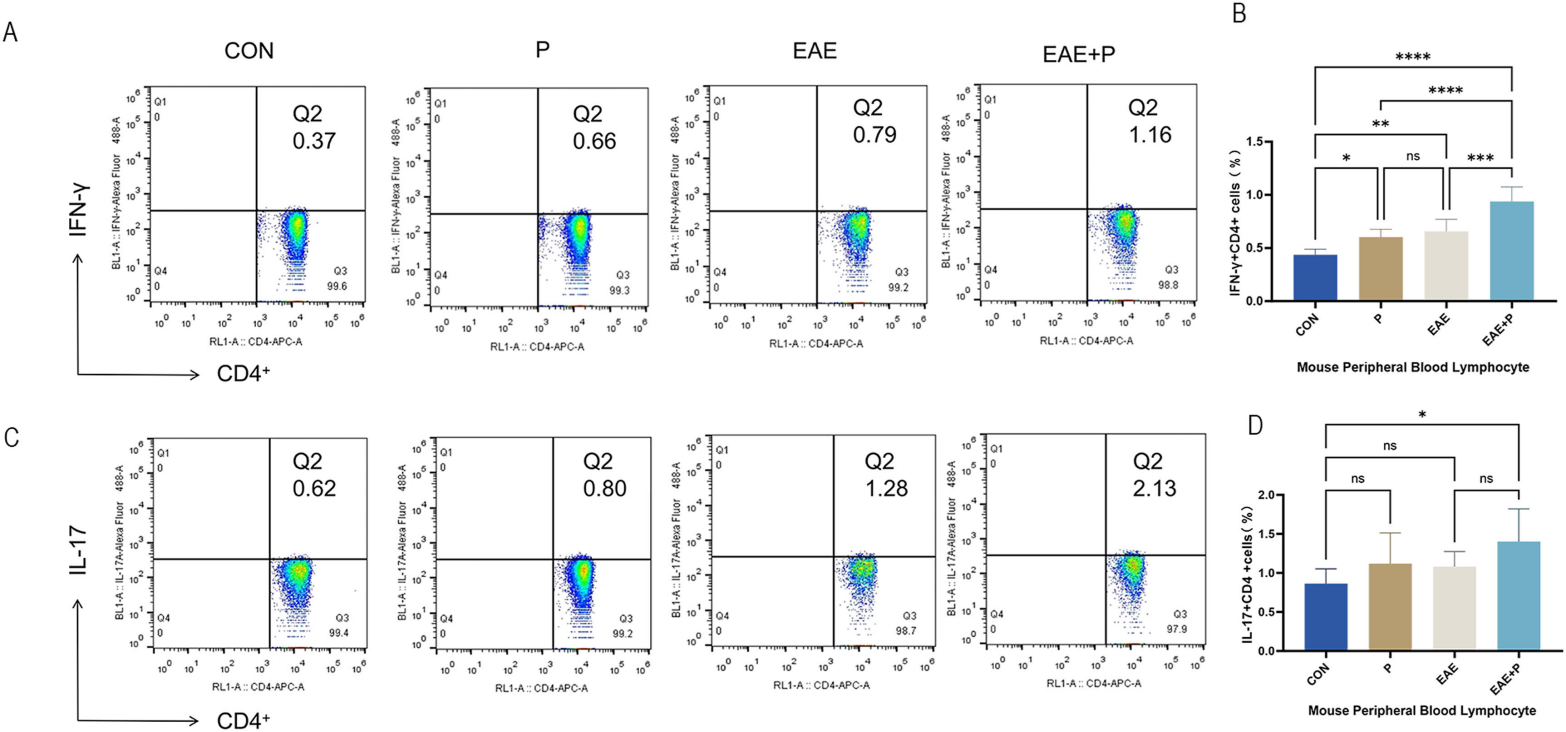
Periodontitis significantly increased the proportion of Th1 cells in the peripheral blood of EAE mice. Proportion of Th1 and Th17 cells in the peripheral blood: **(A)** Frequencies of IFN-γ and IFN-γ-producing CD4^+^ T cells were determined via flow cytometry. Representative dot plots of flow cytometry analysis. **(B)** Analysis of flow cytometry results. **(C)** Frequencies of IL-17 and IL-17-producing CD4^+^ T cells were determined via flow cytometry. Representative dot plots of flow cytometry analysis. **(D)** Analysis of flow cytometry results. One-way ANOVA was used and data are shown as mean ± SD. *p < 0.05, **p < 0.01, ***p < 0.001, ****p < 0.0001, NS p ≥ 0.05.

Since Th1 and Th17 cells play crucial roles in the pathogenesis of EAE, the percentages of Th1 (IFN-γ^+^CD4^+^) and Th17 (IL-17^+^CD4^+^) cells in the peripheral blood were analyzed by flow cytometry to elucidate the potential mechanism by which periodontitis affects the peripheral immune CD4^+^ T cell subsets. The proportion of Th1 (IFN-γ^+^CD4^+^) cells of PBMCs was notably elevated in the P, EAE, and EAE+P groups compared to the CON group (p < 0.01, p < 0.001, and p < 0.0001, respectively). Interestingly, a higher proportion of Th1 cells was observed in the EAE+P group than in the EAE and P groups (p < 0.0001). The proportion of Th1 cells did not significantly differ between the P and EAE groups (**Figures 4A, B**). These results show that both periodontitis and EAE significantly increased the proportion of Th1 (IFN-γ^+^CD4^+^) cells in the peripheral blood, with periodontitis further enhancing this increase during the disease state of EAE mice. The percentage of Th17 (IL-17^+^CD4^+^) cells showed no significant difference between the P and Con groups, nor between the EAE+P and EAE groups. In contrast, it was significantly higher in the EAE+P group than in the Con group (**Figures 4C, D**).

#### 3.2.2 Periodontitis significantly increased the proportion of Th1 cells in the splenic lymphocytes of EAE mice

**Figure 5:**
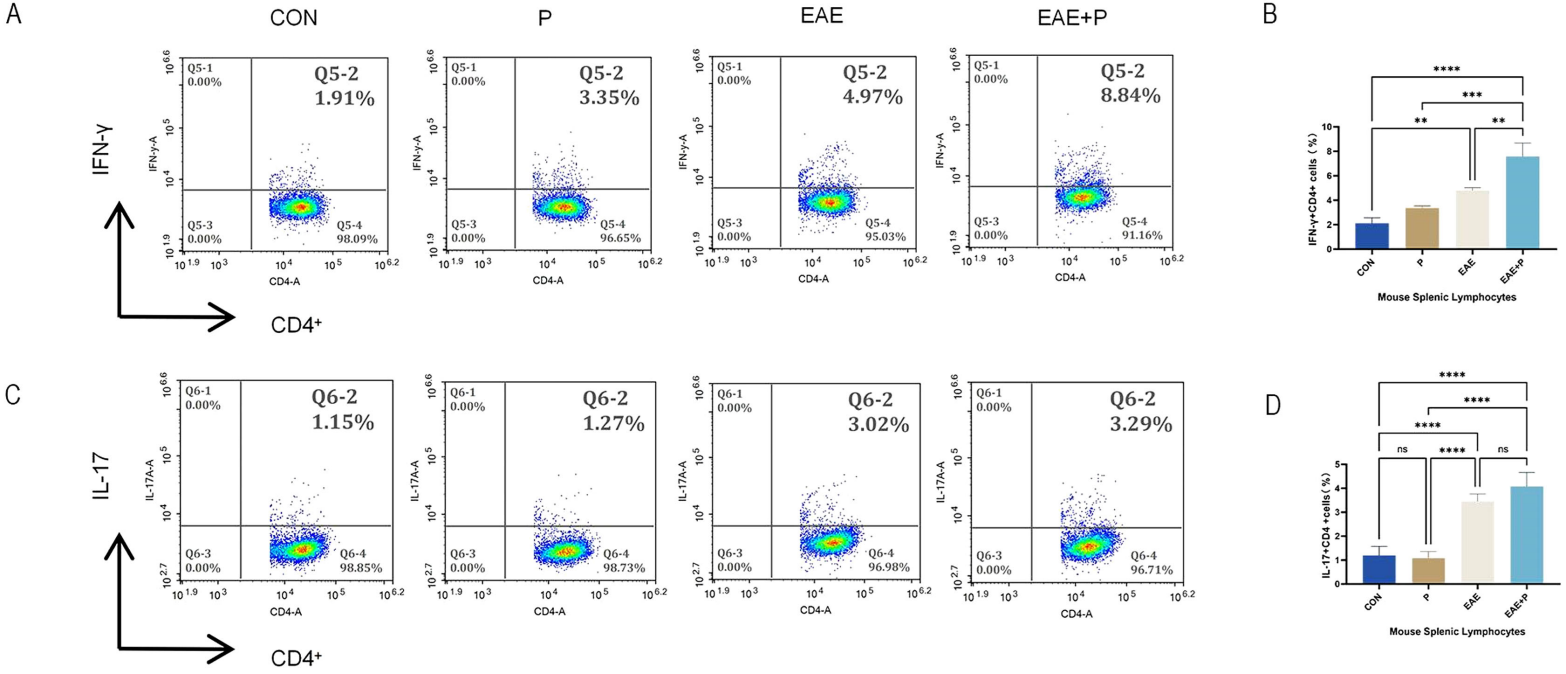
Periodontitis increased the proportion of Th1 cells in the splenic lymphocytes of EAE mice. Periodontitis significantly augmented the propensity of splenocytes in EAE mice to differentiate into IFN-γ^+^CD4^+^ T cell phenotypes. Experimental design: splenocytes from each group were isolated on 17 dpi (the peak of the disease). **(A)** Frequencies of IFN-γ and producing CD4^+^ T cells were determined by flow cytometry. Representative dot plots of flow cytometry analysis. **(B)** Analysis of results of flow cytometry. **(C)** Frequencies of IL-17 and IL-17-producing CD4^+^ T cells were determined by flow cytometry. Representative dot plots of flow cytometry analysis. **(D)** Analysis of flow cytometry results. One-way ANOVA was used and data are shown as mean ± SD. **p < 0.01, ***p < 0.001, ****p < 0.0001,NS p ≥ 0.05.

The spleen plays a critical role in T cell activation, Th1 and Th17 cell differentiation (through antigen presentation), and cytokine modulation. To better understand the mechanism underlying the effect of periodontitis on peripheral immune CD4^+^ T cell activation, the frequency of Th1 (IFN-γ^+^CD4^+^) and Th17 (IL-17^+^CD4^+^) cells in splenic lymphocytes was analyzed using flow cytometry. In the EAE+P group, the ratio of Th1 (IFN-γ^+^CD4^+^) cells was notably elevated as compared to that in the CON, P, and EAE groups (p < 0.01, p < 0.001, p < 0.0001). Moreover, the percentage of Th1 cells in the EAE group was higher than that in the CON group (p < 0.01). However, the P group showed no significant changes compared to the EAE group. These results show that EAE significantly increased the proportion of Th1 (IFN-γ^+^CD4^+^) cells in the splenic lymphocytes of mice, and periodontitis further elevated the proportion of Th1 (IFN-γ^+^CD4^+^) cells in splenic lymphocytes during the disease state in EAE mice. (**Figures 5A, B**) The percentage of Th17 (IL-17^+^CD4^+^) cells in the EAE+P group did not differ significantly from that in the EAE group. Similarly, no significant changes were observed in the P group compared with the CON group (**Figures 5C, D**).

### 3.3 Periodontitis exacerbated disruption of the BBB in the CNS of EAE mice

**Figure 6:**
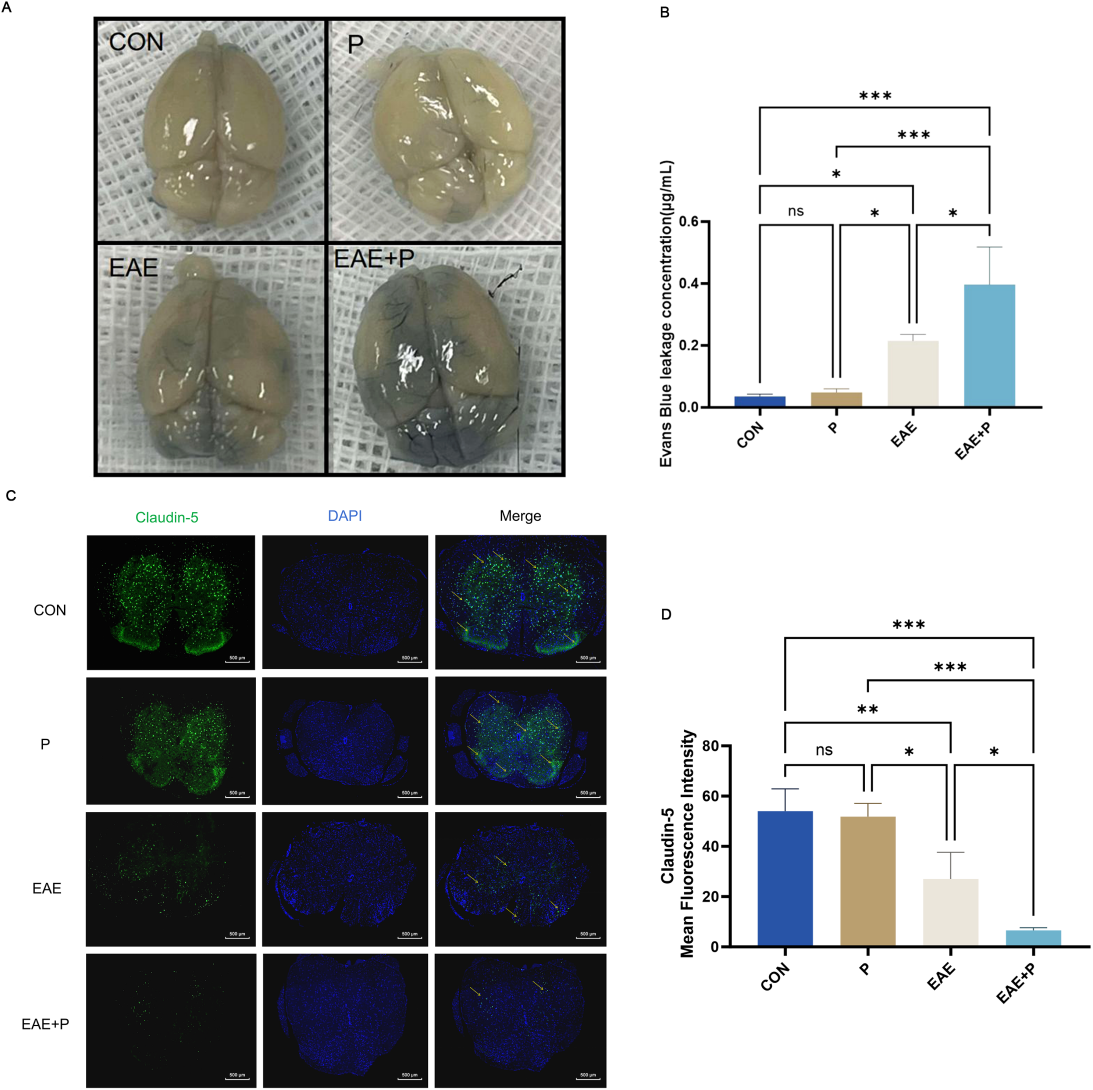
Periodontitis exacerbated disruption of the blood–brain barrier (BBB) in the CNS of EAE mice. Periodontitis significantly exacerbated BBB destruction in EAE mice. At 17 dpi (the peak of EAE), the integrity of the BBB was evaluated through gross measurement of the Evans blue dye extravasation described in the “Materials and Methods” section. **(A)** Representative images of the brain from each group ( n = 5). **(B)** Statistical analysis of the EB dye concentration for each group. **(C)** Immunofluorescent staining of the spinal cord to determine claudin-5 expression, indicated by yellow arrows. **(D)** Statistical analysis of the claudin-5 expression for each group. Data are expressed as mean ± SD. *p < 0.05, **p < 0.01, ***p < 0.001,NS p ≥ 0.05.

Disruption of the BBB was evaluated using the Evans blue dye leakage assay. The concentration of the leaked EB dye was higher in the EAE+P group than in the EAE group (p<0.05). (**Figures 6A, B**) Immunofluorescent staining was used to assess the expression of claudin-5, a critical tight junction protein in the BBB. The expression of claudin-5 in the EAE and EAE+P groups decreased dramatically in the spinal cord relative to that in the CON group (p < 0.01, p < 0.001, respectively), while the P group showed no significant changes. Compared to the EAE group, the expression of claudin-5 in the EAE+P group considerably decreased in the spinal cord (p < 0.05). These results indicate that periodontitis significantly exacerbates BBB disruption in EAE mice (**Figures 6C, D**).

### 3.4 Periodontitis significantly increased the proportion of Th1 cells in the spinal cord of EAE mice

**Figure 7:**
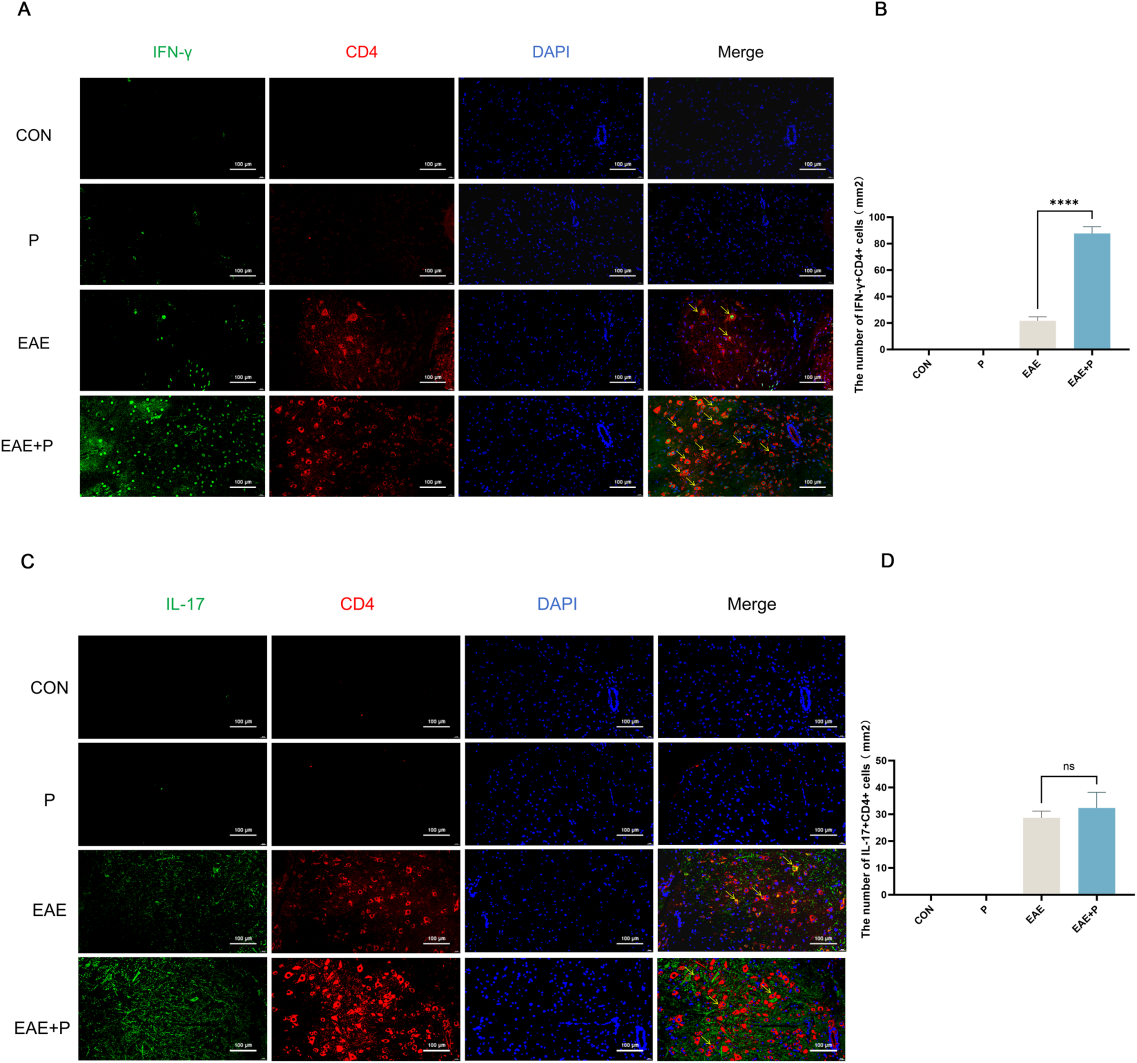
Periodontitis exacerbated Th1 cell infiltration in the CNS of EAE mice. Periodontitis significantly increased inflammatory cell infiltration in the CNS of EAE mice at 17 dpi. **(A)** Quantification of IFN-γ ^+^CD4^+^ T cell expression via immunofluorescent staining of the spinal cord. Yellow arrows indicate IFN-γ^+^CD4^+^-positive cells. **(B)** Number of IFN-γ^+^CD4^+^-positive cells per mm^2^, presented as mean ± SD. Data were derived from five mice per group, with the average number of positive cells from three slides per tissue used for analysis. **(C)** Quantification of IL-17^+^CD4^+^ T cell expression via immunofluorescent staining of the spinal cord. Yellow arrows indicate IL-17^+^CD4^+^-positive cells. **(D)** Number of IL-17^+^CD4^+^-positive cells per mm^2^, presented as mean ± SD. Data were derived from five mice per group, with the average number of positive cells from three slides per tissue used for analysis. ****p < 0.0001,NS p ≥ 0.05.

Immunofluorescent staining of the spinal cord was performed to visualize the expression of IFN-γ^+^CD4^+^ and IL-17^+^CD4^+^ T cells(**Figures 7A-D**). The infiltration of IFN-γ^+^CD4^+^ T cells was significantly increased in the EAE+P group compared to that in the EAE group (p < 0.0001) (**Figure 7B**). These results indicate that periodontitis significantly exacerbated the infiltration of pro-inflammatory cells in the CNS of mice.

### 3.5 Periodontitis enhanced Th1 differentiation and ZAP-70/NF-κB activation in EAE mice

**Figure 8:**
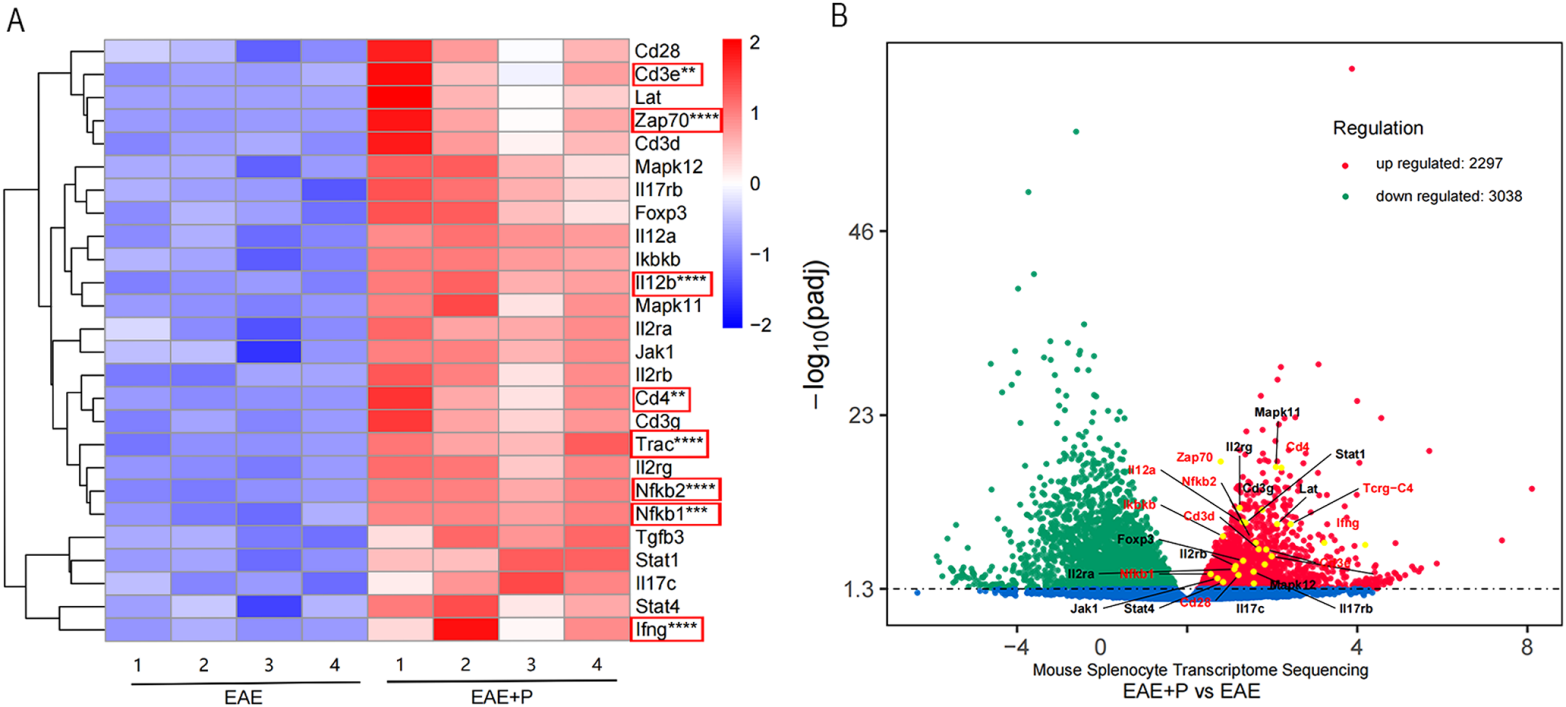
Periodontitis enhanced Th1 differentiation and ZAP-70/NF-κB activation in EAE mice. Periodontitis significantly augmented the propensity of splenocytes from EAE mice to differentiate into IFN-γ^+^CD4^+^ T cell phenotypes. Experimental design: T cells in splenic lymphocytes from each group were isolated at 17 dpi (at the peak of the disease). The gene expression profile of T cell differentiation markers and genes associated with the ZAP70/NF-κB signaling pathway in splenic T cells revealed significant differences between EAE and EAE+P mice. **(A, B)** Spearman correlation heatmap of the 11 significantly different genes. Red denotes a positive correlation, whereas blue implies negative. **p < 0.01, ***p < 0.001, ****p < 0.0001.

To investigate the mechanism by which periodontitis promotes Th1 differentiation in splenic lymphocytes of EAE mice, we performed RNA sequencing on T cells in splenic lymphocytes from both the EAE and EAE+P groups. A total of 5335 genes exhibited significant differential expression between the two groups, including 2297 upregulated and 3038 downregulated genes(**Figure 8B**). Compared to the EAE group, the EAE+P group demonstrated a significant upregulation of Th1 differentiation- and inflammation-related genes, particularly *Ifng* and *Tgfb*. Notably, Th1 differentiation-related genes, such as *Trac, Cd3e, Cd4, Zap70, Nfkb1, Nfkb2*,and *Il-12b* were significantly upregulated in the EAE+P group (**Figures 8A, B**).

**Figure 9:**
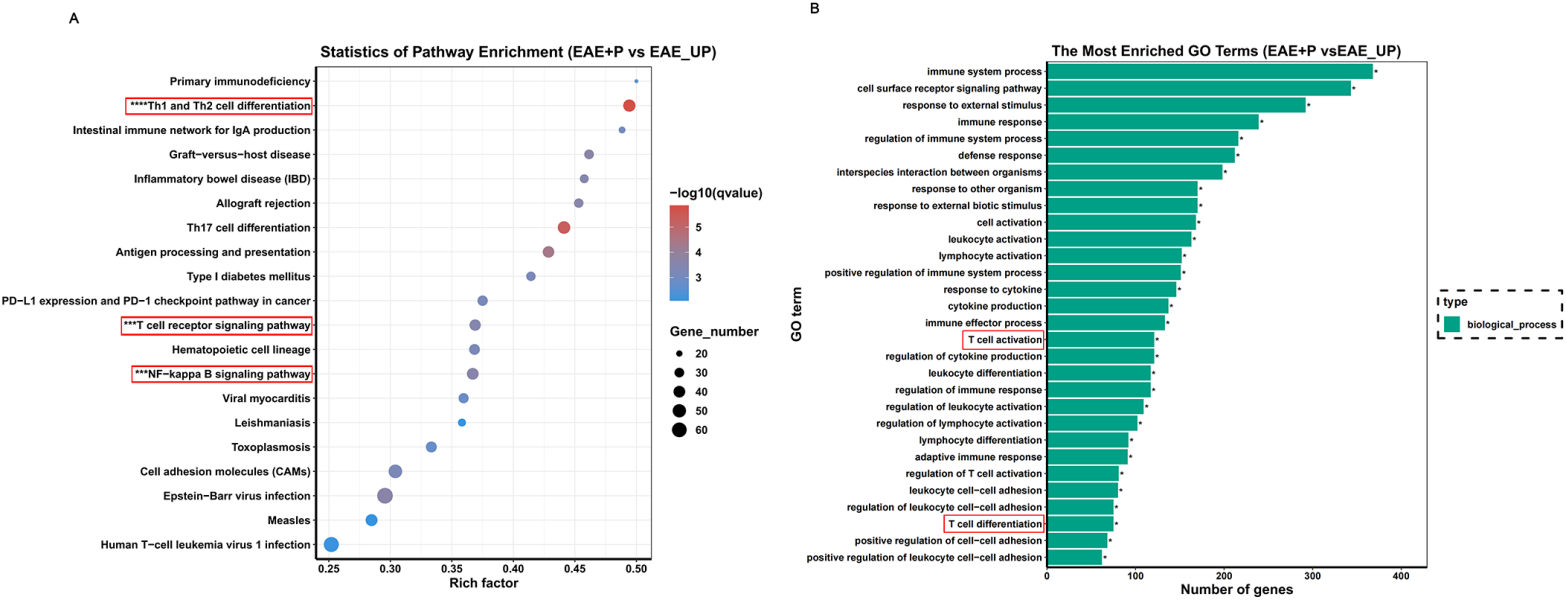
KEGG enrichment analysis based on the gene expression. **(A, B)** KEGG enrichment analysis of differentially expressed genes reveals significant enrichment of Th1 differentiation-associated and NF-κB signaling pathways in the splenic lymphocytes of the P+EAE group relative to the EAE group. This enrichment implies heightened activation or increased expressional activity of these pathways in the P+EAE cohort.

Functional analysis, performed by comparing the results to the KEGG database, revealed that the differentially expressed genes were predominantly associated with the ZAP-70/NF-κB pathway, Th1/Th2 cell differentiation, and T-cell receptor (TCR) signaling pathways. (**Figure 9A**) GO analysis further indicated potential involvement in immune responses, T cell activation, and other related processes (**Figure 9B**).

Based on these sequencing results, we hypothesized that *P. gingivalis* promotes Th1 differentiation in splenic lymphocytes via the ZAP-70/NF-κB signaling pathway. KEGG enrichment analysis of the differentially expressed genes revealed a significant enrichment of Th1 differentiation-associated and NF-κB signaling pathways in the splenic lymphocytes of the EAE+P group compared to the EAE group, suggesting increased activation of these signaling pathways in the EAE+P cohort.

### 3.6 *P. gingivalis* LPS upregulated IFN-γ^+^CD4^+^ T cell differentiation by elevating ZAP70/NF-κB activation in the T cells of splenic lymphocytes

**Figure 10:**
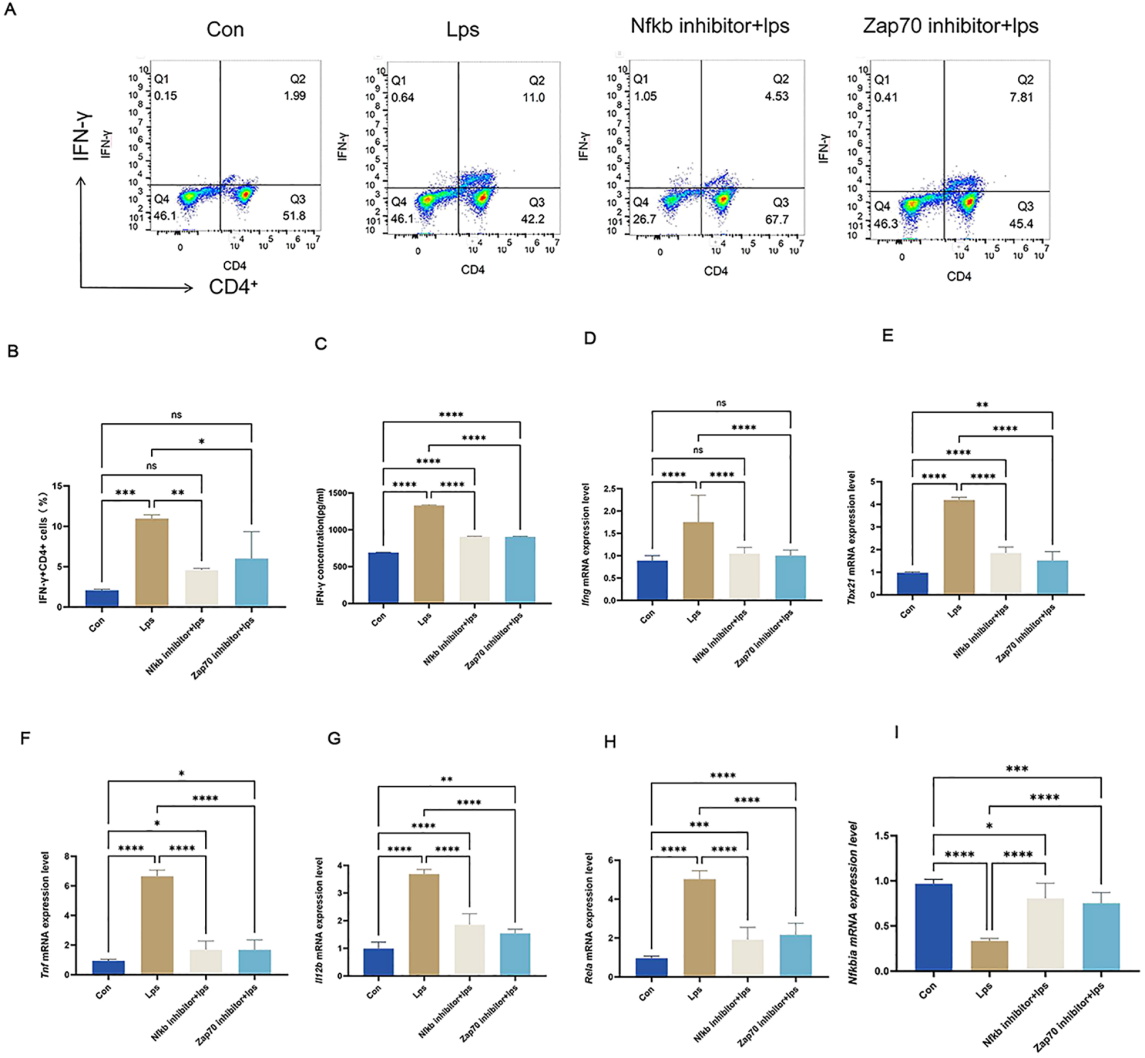
*P. gingivalis* LPS upregulated IFN-γ^+^CD4^+^ T cell differentiation by elevating ZAP70/NF-κB activation in splenic lymphocyte T cells. *P. gingivalis* LPS infection promoted peripheral Th1 cell differentiation in EAE mice via the ZAP70/NF-κB signaling pathway. Experimental design: mouse splenic lymphocytes were pre-treated with ZAP70 and NF-κB inhibitors, stimulated with *P. gingivalis* LPS, and co-cultured *in vitro*. **(A)** Frequencies of IFN-γ and IFN-γ-producing CD4^+^ T cells were determined through flow cytometry. Representative dot plots of flow cytometry analysis. **(B-I)** Analysis of flow cytometry results.Equal amounts of IFN-γprotein were analyzed by elisa. Statistical analysis of the expression of *Ifng*,*Tbx21*,*Tnf*, *lI12b*,*Rela* and *Nfkbia* mRNA. One-way ANOVA was used and data are shown as mean ± SD. *p < 0.05, **p < 0.01, ***p < 0.001,****p < 0.0001,NS p ≥ 0.05.

To further validate the role of *P. gingivalis* in promoting peripheral Th1 cell differentiation, splenic lymphocytes from adult C57BL/6 mice were isolated and subsequently analyzed by flow cytometry (**Figure 10A**). The results (**Figure 10B**) revealed a significant increase in the proportion of IFN-γ^+^CD4^+^ T cells (Th1 cells) after stimulation with *P. gingivalis* LPS. This confirms the pivotal role of *P. gingivalis* in driving the differentiation of Th1 cells, which are key mediators of pro-inflammatory immune responses.

**Figure 11:**
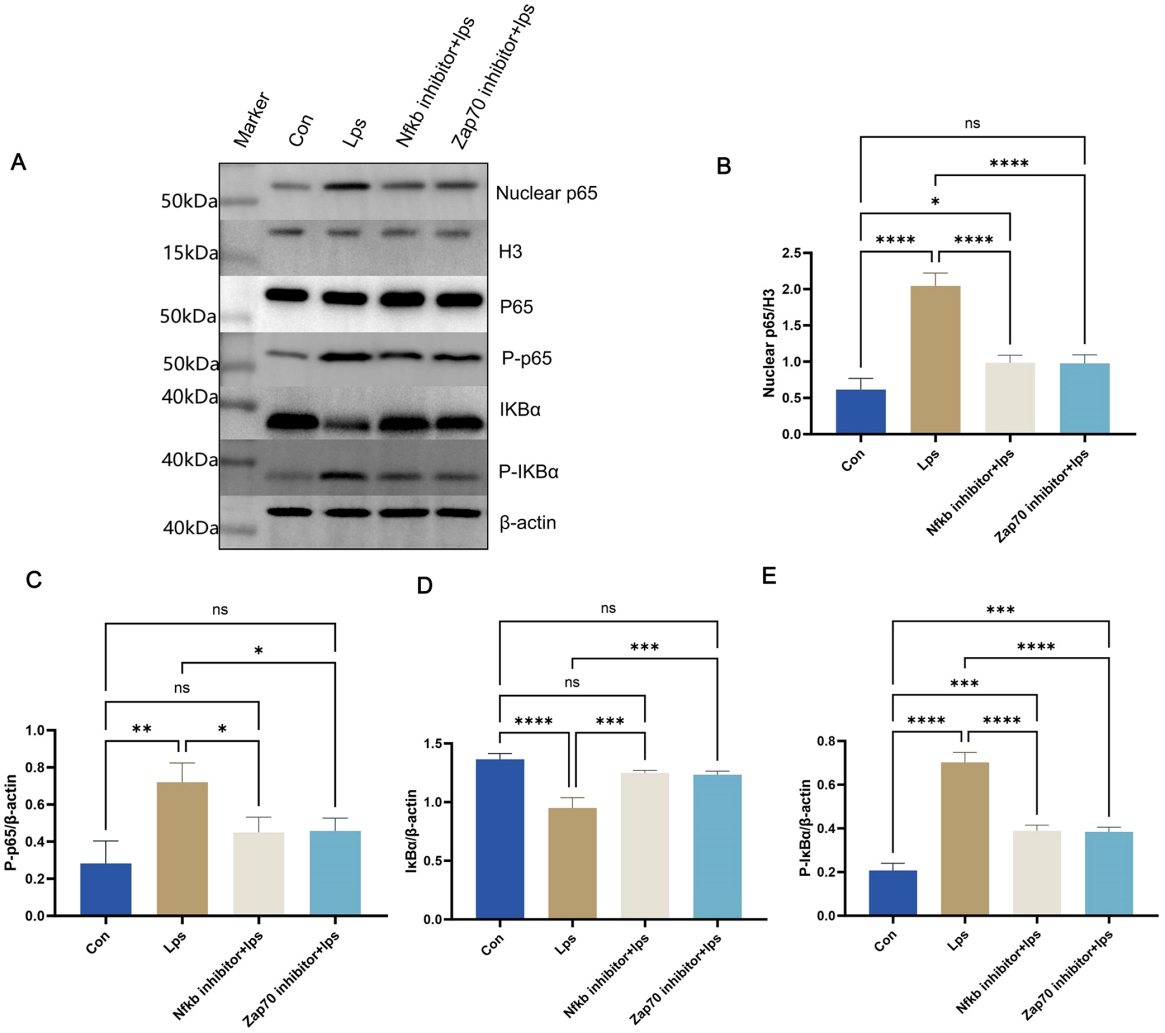
Protein levels were measured in splenic lymphocyte T cells. Equal amounts of protein were analyzed by western blot. Quantitative analysis of nuclear p65, P-p65, IkBα, and P-IkBα in splenic lymphocyte T cells from each group (n = 3). One-way ANOVA was used and data are presented as mean ± SD. *p < 0.05, **p < 0.01, ***p < 0.001, ****p < 0.0001,NS p ≥ 0.05. .

Simultaneously, western blotting results (**Figure 11A**) from T cells in splenic lymphocytes demonstrated enhanced nuclear localization of p65 (**Figure 11B**) and degradation of IkBα protein. (**Figure 11D**) This was accompanied by a significant increase in phosphorylation of p65 (**Figure 11C**) and IkBα (**Figure 11E**) within the NF-κB signaling pathway. These findings indicate robust activation of the NF-κB pathway upon *P. gingivalis* LPS stimulation. The NF-κB pathway is a critical mediator of immune cell activation and differentiation, specifically promoting inflammatory responses.

To verify the crucial role of the NF-κB signaling pathway in *P. gingivalis*-induced Th1 differentiation, Zap70 and NF-κB inhibitors were used. Both inhibitors significantly suppressed the nuclear translocation of p65, degradation of IkBα protein, and phosphorylation of p65 and IκBα (**Figures 11B-E**), effectively inhibiting NF-κB signal activation. Consequently, the differentiation of Th1 cells induced by *P. gingivalis* was markedly reduced, as indicated by a decline in the proportion of IFN-γ^+^CD4^+^ T cells. Additionally, *Tbx21* and *Il12b* expressions were decreased, along with a reduction in the secretion of pro-inflammatory cytokines *Ifng* and *Tnf* (**Figures 10C-I**). This indicates that *P. gingivalis* LPS plays a crucial role in promoting Th1 cell differentiation by activating the ZAP-70/NF-κB signaling pathway, promoting MS associated with periodontitis.

## 4 Discussion

Periodontitis is an infectious disease caused by plaque microorganisms that damage periodontal tissues. It is among the most prevalent chronic oral diseases globally. Severe periodontal disease is the primary cause of tooth loss, affecting approximately 1 billion individuals worldwide (19). Evidence from numerous studies has demonstrated that excessive host immune responses triggered by periodontal pathogens not only lead to inflammation and tissue destruction in periodontal tissue but are also strongly associated with a variety of systemic diseases, including cardiovascular disease, diabetes mellitus, rheumatoid arthritis, and neurodegenerative diseases (20).

*P. gingivalis* is a primary periodontal pathogen. The virulence factors of *P. gingivalis*, including DNA and gingipain, have been identified in the brain and cerebrospinal fluid of patients diagnosed with Alzheimer’s disease (21, 22). Several studies have demonstrated strong correlations between *P. gingivalis* infection, Alzheimer’s disease-related amyloid deposition, and tau protein hyperphosphorylation (22). Furthermore, the inflammatory response triggered by *P. gingivalis* infection and its disruption of the BBB (23) may exacerbate Parkinson’s disease progression. A study by Hu et al. further demonstrated that *P. gingivalis* LPS activates the CNS TLR4/NF-κB pathway, leading to microglial activation and the release of pro-inflammatory cytokines, which worsens neuroinflammation and impairs learning and memory functions (24).

These findings indicate the potential involvement of *P. gingivalis* in neurodegenerative diseases. However, its impact on MS remains unclear. Some studies have indicated that *P. gingivalis* infection may be associated with MS onset (10) and symptom aggravation in mouse EAE models (11). A similar observation was made in present study, where *P. gingivalis* infection intensified the disease process in EAE mice, as evidenced by a significant increase in neurological function, spinal cord inflammation, and demyelination scores during the peak period of EAE. The pivotal role of *P. gingivalis* as a principal periodontal pathogen in neurological disorders is well documented, and a substantial body of evidence indicates that *P. gingivalis* infection markedly influences the peripheral immune system (25). It triggers local inflammation, disrupts tissue barriers, and enables the entry of pro-inflammatory cytokines, outer membrane vesicles, and virulence factors into the bloodstream (20, 26–28). Consequently, dendritic cells, macrophages, and T cells in the peripheral immune system are activated, triggering systemic inflammatory responses and immune imbalances. Our results corroborated these findings, showing a significant increase in the number of Th1 cells in peripheral blood lymphocytes, accompanied by elevated levels of pro-inflammatory factors in mice with periodontitis.

The pathogenesis of EAE is closely associated with the aberrant activation of Th1 and Th17 cells. Th1 cells typically assume a dominant role in the initial stages of the disease, orchestrating an acute inflammatory response in the CNS through the secretion of IFN-γ, which culminates in demyelination and neuronal damage (29, 30). *P. gingivalis* infection further enhances the infiltration of Th1 cells in the spinal cord of EAE mice, and increases the expression of related pro-inflammatory factors. Moreover, Th1 cells play a major role in the peripheral immune responses during EAE (31). In this study, *P. gingivalis* infection significantly enhanced Th1 cell proportion and IFN-γ expression in the peripheral blood and splenic lymphocytes of EAE mice, thereby reinforcing the pro-inflammatory response.

Th17 cells primarily affect the later stages of the disease by maintaining chronic inflammation and driving disease progression (32, 33). The pro-inflammatory role of Th17 cells in the CNS is particularly pronounced. Some EAE studies have suggested that Th1 lymphocytes are the earliest CD4+ T cells to infiltrate the CNS, followed by secondary Th17 cell migration (34). This indicates that a longer observation period may be needed to assess potential changes in Th17 cell populations at later stages of the disease. In the present study, we found that periodontitis did not significantly affect Th17 cells in the peripheral blood, spleen lymphocytes, or spinal cord of either healthy mice or mice with experimental autoimmune encephalomyelitis (EAE) at the peak stage. This is consistent with previous findings. Therefore, we hypothesized that periodontitis escalates EAE mainly through Th1 cells in the peripheral blood, rather than Th17 cells.

*P. gingivalis* has been shown to increase the permeability of the blood–brain and blood–retinal barriers by secreting gingipains and outer membrane vesicles, which disrupt tight junction proteins between endothelial cells, such as the major adhesion proteins of the cerebrovascular endothelium (35). The resultant inflammatory response induces endothelial dysfunction. These findings align with the results of the present study, as *P. gingivalis* infection significantly increased BBB permeability in EAE mice by disrupting claudin-5 (36). Claudin-5, a key transmembrane protein of blood-brain barrier (BBB) tight junctions, directly regulates barrier permeability by forming intercellular channels that restrict the entry of large molecules and harmful substances into the brain. It is primarily expressed in brain microvascular endothelial cells, and its deficiency or dysfunction disrupts BBB integrity, leading to increased nonspecific leakage, such as Evans Blue extravasation. Infiltration of Th1 cells into the spinal cord was also significantly elevated in *P. gingivalis*-infected EAE mice (35). Therefore, *P. gingivalis* infection not only amplifies inflammation in the peripheral and central nervous systems through the activation of Th1 cells but also disrupts the BBB. This allows more Th1 cells and inflammatory factors to enter the CNS, amplifying pro-inflammatory responses and exacerbating demyelination and nerve damage. This local-peripheral-central interaction forms an immunopathological feedback loop across different regions, further escalating the pathological process of EAE.

RNA sequencing confirmed that *P. gingivalis* infection significantly upregulated Th1 differentiation genes in splenic T cells from EAE mice via the ZAP70/NF-κB signaling pathway (13, 37). The differentiation of Th1 cells is co-regulated by several signaling pathways, including JAK-STAT, MAPK, and NF-κB (38–40). Previous studies have shown that NF-κB enhances Th1 cell differentiation by promoting T-bet expression and IFN-γ production, while also amplifying the response of Th1 cells to external stimuli by promoting IL-12 receptor expression. Some researchers indicated that *P. gingivalis* LPS activates the NF-κB signaling pathway in oral epithelial and dental pulp stem cells. LPS, a potent immunostimulatory molecule, activates NF-κB through several upstream pathways, of which the TCR signaling pathway is particularly critical and directly affects T cells. ZAP70 (40, 41) is recruited and activated when the TCR binds to MHC-II, which recognizes antigens and transduces the signal to the intracellular compartment. This process activates the IκB kinase (IKK) complex, whose main catalytic subunit IKKβ phosphorylates IκBα, leading to the ubiquitination and degradation of IκBα, and the release of the p65 subunit of NF-κB (42), which translocates to the nucleus to activate the classical NF-κB pathway. A similar phenomenon was also observed in the present study, where *P. gingivalis* LPS significantly promoted the differentiation of Th1 cells in splenic lymphocytes, increased secretion of the pro-inflammatory factors IFN-γ and TGF-β, and upregulated genes related to Th1 differentiation. Activation of the NF-κB pathway was further evidenced by increased nuclear translocation of the p65 protein, degradation of the IκBα protein, and elevated phosphorylation. Based on these observations, we suggest that *P. gingivalis* mediates Th1 cell differentiation through the ZAP70/NF-κB signaling pathway, thereby exacerbating the course of EAE.

To validate the effect of *P. gingivalis* on the ZAP70/NF-κB signaling pathway in Th1 cell activation, we conducted *in vitro* experiments using splenic T cells stimulated by *P. gingivalis* LPS. The results showed that *P. gingivalis* LPS activated the NF-κB signaling pathway and promoted Th1 cell differentiation. The use of the inhibitor ZAP180013, which competes with the ATP-binding site of ZAP70 kinase to prevent ZAP70 activation and block downstream TCR signaling, inhibited LPS-stimulated p65 nuclear translocation and phosphorylation. This effectively blocked the NF-κB signaling pathway and restored Th1 cell levels to those of the Con group. The secretion of pro-inflammatory factors and expression of Th1 differentiation-related genes were also significantly reduced. Similarly, after inhibiting the degradation of IκB protein using PDTC, we observed partial inhibition of LPS-stimulated p65 nuclear translocation and complete suppression of p65 phosphorylation. This blocked the NF-κB pathway and partially reversed the proportion of Th1 cells as well as the expression of TNF-α, IFN-γ, and IL-12. These results suggest that the activation of the ZAP70/NF-κB signaling pathway significantly promotes the differentiation of Th1 cells and the secretion of pro-inflammatory factors under *P. gingivalis* LPS stimulation. Inhibition of p65 further demonstrated that ZAP70 is a critical upstream regulator of the NF-κB signaling pathway under *P. gingivalis* LPS stimulation, playing a key role in Th1 cell differentiation. Therefore, we conclude that *P. gingivalis* LPS promotes Th1 cell differentiation through the ZAP70/NF-κB signaling pathway.

To the best of our knowledge, this study is the first to demonstrate EAE progression accelerated by periodontitis while elucidating its underlying mechanisms. Through activation of the ZAP70/NF-κB signaling pathway by *P. gingivalis* LPS, periodontitis promoted the differentiation of peripheral blood Th1 cells, increased the permeability of the BBB, and enhanced inflammatory infiltration and demyelination of the CNS, intensifying the severity of EAE. These findings highlight that periodontitis is not only manifested by local oral inflammation but also significantly amplifies CNS inflammation, providing direct evidence of the relationship between *P. gingivalis* infection and encephalopathy.

Our results highlight periodontitis as a critical risk factor for MS. Therefore, prevention and control of periodontitis may be effective in reducing further deterioration of MS. Periodontal health management should be considered an important component of systematic disease management for patients with MS. The potential benefits include mitigated CNS inflammation and reduced risk of demyelinating lesions. ZAP70/NF-κB inhibitors may be a potential therapeutic target for the treatment of *P. gingivalis*-mediated Th1 cell-related diseases, such as EAE, angiotensin II-dependent hypertension, and atherosclerosis. Future studies must explore the ZAP70/NF-κB signaling pathway and molecular mechanisms by which periodontitis exacerbates EAE *in vivo*.

## 5 Conclusion

*P.gingivalis* drives Th1 cell differentiation through the ZAP70/NF-κB pathway, intensifying EAE progression and promoting demyelination. The infection also increases the BBB permeability. Targeting the ZAP70/NF-κB signaling pathway effectively suppresses Th1 cell activation, providing a potential therapeutic target.

## Data Availability

Raw data have been deposited to National Center for Biotechnology Information (NCBI) under the BioProject number PRJNA1233427.
