# Supplementary figures and images for: Porphyromonas gingivalis exacerbates experimental autoimmune encephalomyelitis by driving Th1 differentiation via ZAP70/NF-κB signaling
Source: Front Immunol. 2025 Mar 18;16:1549102. doi: 10.3389/fimmu.2025.1549102 (PMC11958167; doi:10.3389/fimmu.2025.1549102)

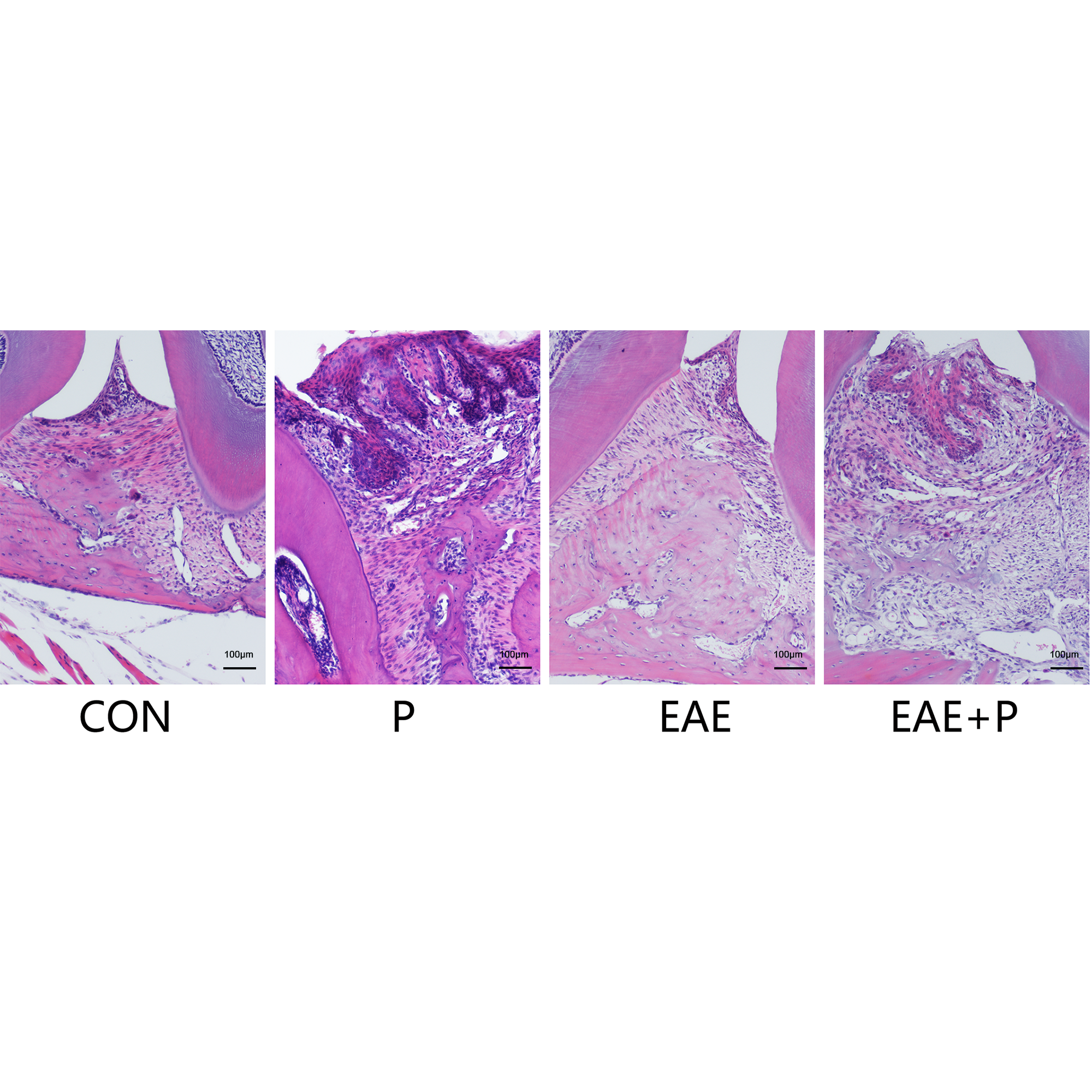

Supplement: Supplementary file 1 [file Image1.tif]
